# Associations between body mass index and episodic memory for recent eating, mindful eating, and cognitive distraction: A cross‐sectional study

**DOI:** 10.1002/osp4.728

**Published:** 2024-01-04

**Authors:** Elanor C. Hinton, Victoria Beesley, Sam D. Leary, Danielle Ferriday

**Affiliations:** ^1^ NIHR Bristol Biomedical Research Centre Diet and Physical Activity Theme University of Bristol Bristol UK; ^2^ Nutrition and Behaviour Unit School of Psychological Science University of Bristol Bristol UK

**Keywords:** adults, appetite, body mass index, cognitive distraction, eating behaviors, episodic memory for recent eating, mindful eating, psychology

## Abstract

**Objectives:**

Eating while distracted has been associated with a higher body mass index (BMI), whereas mindful eating and episodic memory for recent eating have shown the opposite pattern. This pre‐registered, global study (https://osf.io/rdjzk) compared the relative association between these variables (and four “positive controls”: restraint, disinhibition, emotional eating, plate clearing) and self‐reported BMI. The timing of data collection (April–May 2020) during the SARS‐CoV‐2 pandemic enabled an investigation of the impact of stay‐at‐home restrictions imposed on the UK population on the measures of eating behavior.

**Methods:**

An online survey was completed, including: (i) demographic data (e.g., self‐reported BMI), (ii) Likert ratings assessing episodic memory for recent eating, mindful eating, cognitive distraction, restrained eating, emotional eating, disinhibition and plate clearing over the last 12 months and the last 7 days (during the first UK COVID‐19 lockdown), and (iii) the Mindful Eating Questionnaire (MEQ).

**Results:**

A large adult sample participated (*N* = 846; mean (SD) age = 33.0 (14.3) years; mean (SD) BMI = 24.6 (5.6) kg/m^2^). Mindful eating (MEQ‐total score) was associated with a lower self‐reported BMI (*β* = −0.12; 95% CI = −0.20, −0.04; *p* = 0.004), whereas disinhibited eating was associated with a higher self‐reported BMI (*β* = 0.30; 95% CI = 0.21, 0.38; *p* < 0.001). In UK participants (*n* = 520), consistent changes in eating behavior during lockdown were not found. For those that did experience change, decreases were reported in; emotional eating, disinhibited eating, focusing on taste during a meal (a measure of mindful eating), and using a smart phone while eating.

**Conclusions:**

These findings provide evidence in a large global sample for associations between BMI and (i) mindful eating, and (ii) disinhibited eating. Future research should evaluate whether mindful eating demonstrates a prospective association with body weight and should consider mechanisms of action.

## 1 INTRODUCTION

One area that has received considerable attention in laboratory‐based research is the role of attentive and memory‐based processes in the control of human food intake. For example, there is evidence that episodic memory of recent eating plays an integral role in determining both immediate and future food consumption.^1^, ^2^, ^3^, ^4^, ^5^, ^6^, ^7^, ^8^, ^9^, ^10^ Food intake is reduced if people are asked to recall details of a recent meal,^2^ and altering the initial formation of the memories by either increasing the attention paid to food or by disrupting attention paid to food, increases and decreases food intake, respectively.^4^, ^8^, ^9^, ^11^, ^12^ Specifically, attention to food can be disrupted by distracting people from a meal or snack, and indeed there is a body of evidence which demonstrates that eating while using a smartphone,^13^, ^14^ watching television^15^ or playing a computer game^16^ leads to greater food intake. Mindful eating, on the other hand, encourages a greater focus on the sensory properties of foods (among other components) and there is also evidence to suggest that greater mindful eating reduces food intake.^17^, ^18^, ^19^ However, this body of research has relied predominantly on acute manipulations of memory for recent eating, distracted eating and mindful eating and either the immediate or delayed measurement of energy intake (typically no longer than 3 h post‐manipulation) in laboratory settings. In addition, we note that there have been some failures to observe the effects of manipulating memory for recent eating, distracted eating and mindful eating on acute measures of food intake in the literature.^20^, ^21^, ^22^, ^23^, ^24^

An additional concern with these laboratory‐based observations is whether there would be “compensatory eating” for any acute effects over subsequent days/weeks/months. In part to address this concern, increasing research is focusing on establishing evidence for potential chronic effects of the aforementioned cognitive controls of food intake (episodic memory for recent eating, mindful eating, and distracted eating) by assessing associations with longer‐term measures of dietary behavior (e.g., body mass index, BMI^25^). There have been reports of episodic memory deficits in obese individuals,^7^, ^26^ mindful eating has been associated with lower BMI,^27^, ^28^, ^29^, ^30^, ^31^ whereas a positive relationship between distracted eating (e.g., screen time) and BMI^32^, ^33^ has been reported. To date, studies exploring the relationship between these cognitive controls of food intake and/or BMI have tended to investigate factors in isolation (e.g., a sole focus on mindful eating and BMI), leading to difficulty comparing the *relative* strength of the associations between each measure with BMI.

Consequently, the main aim of this online global study was to investigate the relationship between cognitive controls of food intake (i.e., episodic memory for recent eating, mindful eating, and cognitive distraction) and self‐reported BMI in a large sample. It was hypothesized that BMI would be negatively associated with episodic memory for recent eating and mindful eating, but positively associated with cognitive distraction. The aim was to compare the relative strength of the association of each of these variables with BMI. To establish the validity of the findings, this study also incorporated other psychological variables that have consistently been associated with BMI as “positive controls.”^34^ Psychological variables that have been positively associated with BMI are restrained eating (the intention to restrict food intake to prevent body weight gain),^25^ emotional eating (consuming food due to feeling negative emotions),^25^ disinhibited eating (the tendency to overeat in response to various stimuli),^25^ and plate clearing (the tendency to clear one's plate when eating).^35^

The data collection for this study coincided with the first wave of the SARS‐CoV‐2 pandemic lockdown in the UK (April–May 2020) and stringent stay‐at‐home restrictions were imposed on the UK population. Consequently, these wider circumstances represented a unique opportunity to explore the impact of stringent stay‐at‐home restrictions on the measures of cognitive controls of food intake (i.e., memory for recent eating, mindful eating, and distracted eating) and the positive controls (restrained eating, emotional eating, disinhibited eating, and plate clearing). Indeed, these data add to a growing body of literature exploring changes in eating behaviors experienced during COVID‐19 lockdowns. Previous studies exclusively employ an online survey methodology in both global samples^36^, ^37^ and samples from individual countries.^38^, ^39^ To the authors' knowledge, no studies to date have explored the effects of lockdown restrictions on cognitive controls of food intake. However, there are reports demonstrating that screen time increased during COVID‐19 restrictions.^40^ It was predicted that lockdown during the COVID‐19 pandemic would result in changes in individuals reported eating behaviors, such as greater frequency of eating while distracted or while bored, compared to before the pandemic. To assess this hypothesis, questions assessing memory for recent eating, mindful eating, distracted eating, and the positive controls were asked twice: once where participants considered usual dietary behavior over the last 12 months, and again where participants considered the last 7 days (during the first lockdown in the UK).

## 2 METHODS

### 2.1 Participants

Data were collected between 10 April and 10 May 2020, during the first national lockdown for COVID‐19 in the UK. An online convenience sampling approach was taken, whereby recruitment was via advertisements posted on social media platforms and via the online recruitment tool Prolific Academic. Participants were informed that the purpose of the study was to explore the relationship between eating behavior and body weight. All participants gave informed consent. Participants voluntarily entered into a prize draw to win a £50 Amazon voucher or were paid £3.38 upon survey completion if recruited by Prolific Academic. Our inclusion criteria for participants were as follows: over the age of 18 and not currently eating (since eating while completing the questionnaire might have distracted the participant or biased the responses). This study was approved by the School of Psychology Research Ethics Committee, University of Bristol (Checklist 101143).

### 2.2 Questionnaire and procedure

The design and statistical analysis plans for this survey were pre‐registered via the Open Science Framework (https://osf.io/s9dvj/). The original questionnaire was designed in combination with a Master's thesis and included additional questions beyond the scope of this paper. To address all the pre‐registered hypotheses, those not included below (analyses of the Adult Eating Behavior Questionnaire food approach and avoidance subscales and bespoke questions regarding hoarding, eating in the absence of hunger, and eating with others) are included in Supporting Information S1 for interested readers.

The questionnaire was designed and distributed via the behavioral experiment platform Gorilla (https://gorilla.sc/). Participants accessed the online survey via a link and were then invited to read an information sheet outlining the inclusion criteria and the study aims. Consent to participate was confirmed online. The main survey comprised eight different sections of questions presented to participants in the same order. First, demographic data (sex, age, ethnicity, nationality, highest level of education completed, average monthly take‐home household income, country, height, weight and whether the participant had previously been diagnosed with an eating disorder) were collected—see Table S1 for precise wording of questions. Second, participants reported the time since they last ate or drank, how vivid their memory was for that eating episode, and appetite ratings using 100‐point visual analog scales with anchors “Not at all” and “Extremely.”^20^ Third, participants were asked to reflect upon how they usually eat over the last 12 months and provide Likert ratings about the frequency of episodic memory for recent eating, restrained eating, emotional eating (eating when sad, stressed, bored, or to feel better), disinhibition and plate clearing (for precise wording of questions in sections three to six see Table 2). Fourth, participants were asked to reflect upon their usual evening meal over the last 12 months and provide Likert ratings about the frequency of mindful eating (taking a moment to appreciate the taste, smell and texture of food) and distracted eating (eating while looking at phone, playing a computer game or watching TV). Respectively, in the fifth and sixth section, participants repeated the measures about how they usually eat from section three and reflecting upon their usual evening meal from Section 4 but considering the last 7 days. Section seven contained the Mindful Eating Questionnaire (MEQ^31^), and section eight contained questions related to SARS‐CoV‐2 (current symptoms or diagnosis, and about symptoms in the household over the past 7 days). With the exception of the demographic questions, the order of questions within each section was randomized. At the end of the survey, participants were given further information about the aim and purpose of the study.

### 2.3 Body mass index

BMI was calculated as participant self‐reported body weight in kilograms (kg) divided by participant self‐reported height in meters squared (m^2^). Previous research has shown self‐reported height, body weight, and BMI to be highly correlated with measured variables.^41^

### 2.4 Statistical analysis

*Data screening and composition of scores*: The raw data were screened and participants excluded if they were under the age of 18 or currently eating. Data were also screened based on time taken to complete the survey, with data removed that were completed too quickly (suggesting lack of attention) or too slowly (suggesting participants did not complete in one sitting). Inspection of the BMI data revealed some impossible values where participants had entered height or body weight incorrectly. *Z*‐scores were calculated for both BMI and time taken to complete the study, in which *z*‐score values less than −3.29 and more than 3.29 were removed.^42^

A composite score was calculated to represent distracted eating. This comprised the mean response to the questions regarding the extent of eating while gaming, using a smart phone, and watching TV. In a deviation from our pre‐registration, it is noted that the distraction composite score originally included a rating about frequency of eating with others. However, due to the wealth of literature on social facilitation effects of eating,^43^ it was subsequently decided that to consider eating with others merely as a distractor was to oversimplify social effects on eating behavior and this measure was therefore removed from the calculation of the composite distractor score. For interested readers, the relationship between eating with others and self‐reported BMI is reported in the Table S9. Another composite score was calculated to represent emotional eating, which comprised the mean response to the questions regarding eating when sad, bored, stressed, or to feel better.

Histograms for each variable were visually inspected for outliers and the data were checked for normality using skewness and kurtosis values. Finally, Cronbach's alpha was calculated for the MEQ to establish the internal consistency in the present study. Data analysis was conducted using IBM SPSS Statistics v26 for Windows.

*Sample descriptive statistics and demographics*: The frequency (absolute and percentage) of sociodemographic variables and responses to the bespoke eating behavior questions over the last 12 months and the last 7 days were calculated. Means and standard deviations of validated questionnaire scores and appetite ratings were also calculated.

*Regression models*: To investigate the main aim of whether the three cognitive control of food intake variables (over past 12 months) (episodic memory for recent eating, mindful eating [MEQ total score] and distracted eating composite score) and four positive controls (restrained eating, emotional eating composite score, disinhibition, plate clearing) were associated with BMI, a two‐phased approach was taken using forced entry multiple regression models separately for each eating behavior variable. First, in minimally adjusted models, age and sex were included as confounding variables. Secondly, in fully adjusted models, education and monthly household income were included in addition to age and sex. In alignment with Robinson et al.,^35^ these confounders were chosen due to known relationships with BMI.^44^ Standardized beta coefficients are presented as the eating behavior variables are not in the same units of measurement. To check for multicollinearity in the regression models, variance inflation factors (VIFs) were calculated.

Further regression models were conducted, which included all seven eating behavior measures described above, both minimally and fully adjusted for confounders. This allowed comparison of the standardized beta coefficients for eating behavior variables, and calculation of the combined explained variance of all the predictors of BMI.

If the total MEQ score was found to be associated with BMI, the subscales of the MEQ would then be compared using standardized beta weights in individual regression models (see Table S5). An exploratory analysis was also conducted to compare the association between different types of distraction while eating (i.e., TV watching, playing a computer game, and being on the phone) and different types of emotional eating (i.e., eating when sad, bored, stressed and to feel better) and BMI (seeTable S5).

*Before and after lockdown analyses*: To determine whether differences in eating behavior were reported during the lockdown period, a series of crosstabulations were performed using the McNemar–Bowker test of symmetry for the seven eating behavior variables in turn.^45^ These statistical tests compared responses to the bespoke questions regarding individuals' typical eating behavior “pre‐lockdown” (previous 12 months) and “during lockdown” (previous 7 days). This analysis was restricted to UK participants only as participants from other countries may have been subject to different restrictions at the time of data collection. The McNemar–Bowker test is an omnibus test and a significant result indicates that the contingency table is not symmetric but does not reveal where the differences lie (i.e., whether the behaviors increased or decreased in frequency). Therefore, to interpret the extent to which behaviors increased or decreased across the two timepoints, for each individual and for each eating behavior variable in turn, the shift in responses (increase, decrease or no change) between the last 12 months and last 7 days was calculated. An inspection of the data revealed that for many of the variables, a similar proportion of participants increased and decreased the behavior. Therefore, to determine the eating behavior variables where a consistent change in one direction (i.e., either a predominant increase or decrease in that behavior across the sample) was observed, an arbitrary threshold was set to establish “meaningful change.” This threshold was set as those variables which showed a difference of 10% points between mean increase and decrease.

## 3 RESULTS

### 3.1 Data screening

Nine hundred and nine participants participated in the survey, but three were excluded as aged under 18, 36 were currently eating and 24 completed the survey too slowly, which left 846 for analysis.

The final sample (*N* = 846) had a mean (SD) age of 33.0 (14.3) years (range 18–84) and a mean BMI (SD) of 24.6 (5.6) kg/m^2^ (range 14.5–52.8). The sample was predominantly female (69%) and white (82%), with 55% of participants identifying themselves as British, and the majority having a university degree (63%). 62% of participants were currently residing in the UK, with other participants from across the globe (see Figure S1). Five participants had a COVID‐19 diagnosis and 31 had experienced COVID‐19 symptoms within the past 7 days. The survey took a mean (SD) of 1396 (2837) min to complete. The full list of sample characteristics is shown in Table 1. Table S2 reports the mean appetite ratings and state questions at the time of questionnaire completion.

**TABLE 1:** Participant sociodemographic characteristics.

Characteristic	*n* (% in each category)
Sex	
Female	584 (69.0%)
Male	258 (30.5%)
Prefer not to say	4 (0.5%)
Self‐reported BMI (kg/m^2^)	
<18.5, underweight	64 (7.6%)
18.5–24.9, healthy	475 (56.1%)
≥25–29.9, overweight	176 (20.8%)
≥30, obese	131 (15.5%)
Level of education	
None	10 (1.2%)
GCSEs or equivalent	67 (7.9%)
A‐levels or equivalent	215 (25.4%)
Degree	304 (35.9%)
Higher degree	226 (26.7%)
Prefer not to say	24 (2.8%)
Monthly household income (£)	
<899	136 (16.1%)
900–1149	71 (8.4%)
1150–1549	90 (10.6%)
1550–1849	52 (6.1%)
1850–2099	29 (3.4%)
2100–2399	54 (6.4%)
2400–2799	48 (5.7%)
2800–3399	56 (6.6%)
3400–4000	56 (6.6%)
>4001	120 (14.2%)
Prefer not to say	134 (15.8%)
Ethnicity	
Asian or Asian–British Bangladeshi	2 (0.2%)
Asian or Asian–British Indian	35 (4.1%)
Asian or Asian–British Pakistani	6 (0.7%)
Black or Black–British Caribbean	7 (0.8%)
Black or Black–British African	18 (2.1%)
Chinese	11 (1.3%)
Mixed White and Asian	16 (1.9%)
Mixed White and Black African	7 (0.8%)
Mixed White and Black Caribbean	4 (0.5%)
White	696 (82.3%)
Other	30 (3.2%)
Prefer not to say	10 (1.2%)
Unknown	3 (0.4%)
Nationality	
African	33 (3.9%)
Asian	55 (6.5%)
British	467 (55.2%)
European	208 (24.6%)
North American	50 (5.9%)
South American	20 (2.4%)
Other	13 (1.5%)
No. in household	
0	68 (8.0%)
1	166 (19.6%)
2	141 (16.7%)
3	180 (21.3%)
4	78 (9.2%)
5>	65 (7.7%)
Missing^a^	148 (17.5)
History of eating disorder	
Yes	41 (4.8%)
No	805 (95.2%)

*Notes*: *Nationality Other:* Albanian (*n* = 1), Australian (*n* = 3), Non‐British White (*n* = 1), Unknown (*n* = 1). *Ethnicity Other:* 30 participants described themselves as the following ethnicities: Arab (*n* = 5), Asian–Nepalese (*n* = 2), Hispanic (*n* = 10), Latin (*n* = 6), and Mixed (*n* = 8).

Abbreviation: BMI, body mass index.

^a^ Missing values since the question was added after the survey had begun.

### 3.2 Typical eating behaviors over the last 12 months

The frequency (absolute and percentage) of responses to the bespoke eating behavior questions can be found in Table 2. Eating an evening meal was often accompanied by the use of a smart phone or watching television, whereas eating an evening meal while playing a computer game was much less frequently reported. Few people regularly reported thinking about their last meal when about to eat again.

**TABLE 2:** Frequency (absolute and percentage) of responses to the bespoke eating behavior questions ($ denotes variable used in main analysis).

		When thinking about the last 12 months					When thinking about the last 7 days				
		Never	Rarely	Sometimes	Mostly	Always	Never	Rarely	Sometimes	Mostly	Always
Episodic memory for recent eating$											
How often do you think of your last meal when you are about to eat again?	Freq.	208	333	201	70	34	242	298	185	83	38
	%	24.6	39.4	23.8	8.3	4.0	28.6	35.2	21.9	9.8	4.5
Mindful eating											
How often do you take a moment to appreciate the:											
Taste of your food?	Freq.	11	104	265	317	150	35	113	262	291	145
	%	1.2	11.4	29.2	34.9	16.5	4.1	13.4	31.0	34.4	17.1
Smell of your food?	Freq.	59	230	268	197	93	85	214	236	209	102
	%	6.5	25.3	29.5	21.7	10.2	10.0	25.3	27.9	24.7	12.1
Texture of your food?	Freq.	95	226	255	196	75	125	231	228	178	84
	%	10.5	24.9	28.1	21.6	8.3	14.8	27.3	27.0	21.0	9.9
	%	1.1	12.2	20.5	44.0	15.4	8.9	10.5	12.3	31.3	37
Distracted eating$											
During your usual evening meal, how often do you:											
Eat while looking at your phone?	Freq.	166	249	272	133	26	281	222	189	115	39
	%	19.6	29.4	32.2	15.7	3.1	33.2	26.2	22.3	13.6	4.6
Play a computer game when eating?	Freq.	614	135	75	20	2	675	81	62	24	4
	%	72.6	16.0	8.9	2.4	0.2	79.8	9.6	7.3	2.8	0.5
Watch television while eating?	Freq.	94	149	254	273	76	163	128	199	234	122
	%	11.1	17.6	30.0	32.3	9.0	19.3	15.1	23.5	27.7	14.4
Restrained eating											
How often do you avoid eating tempting foods?	Freq.	12	155	407	232	41	73	265	329	161	18
	%	1.3	17.1	44.8	25.5	4.5	8.6	31.3	38.9	19.0	2.1
Emotional eating											
How often do you eat:											
When you are sad?	Freq.	47	188	352	221	39	161	275	259	115	36
	%	5.2	20.7	38.7	24.3	4.3	19.0	32.5	30.6	13.6	4.3
When you are stressed?	Freq.	80	215	277	233	42	135	273	228	166	44
	%	8.8	23.7	30.5	25.6	4.6	16.0	32.3	27.0	19.6	5.2
When you are bored?	Freq.	67	269	354	123	34	70	187	325	217	47
	%	7.4	29.6	38.9	13.5	3.7	8.3	22.1	38.4	25.7	5.6
To make yourself feel better?	Freq.	33	159	330	231	94	117	248	318	138	25
	%	3.6	17.5	36.3	25.4	10.3	13.8	29.3	37.6	16.3	3.0
Disinhibition											
How often do you find it hard to control the amount of food you consume?	Freq.	54	159	325	236	73	91	290	289	130	46
	%	5.9	17.5	35.8	26.0	8.0	10.8	34.3	34.2	15.4	5.4
Plate clearing											
How often do you leave food on your plate?	Freq.	19	67	153	418	190	261	352	161	55	17
	%	2.1	7.4	16.8	46.0	20.9	30.9	41.6	19.0	6.5	2.0

The MEQ total score was used to assess the association between mindful eating and BMI. Table 3 reports the mean and reliability of the MEQ total and subscale scores for this sample. The internal consistency of the MEQ total score and subscales of disinhibition and awareness were very good, whereas the external and distracted eating subscales fell below the acceptable range.^46^

**TABLE 3:** Mean (SD) and reliability of the MEQ.

Measure	Mean	SD	95% CI (lower, upper)	Internal consistency (a)
MEQ total	2.78	0.33	2.76, 2.80	0.8
MEQ disinhibition	2.71	0.57	2.66, 2.74	0.8
MEQ awareness	2.60	0.59	2.56, 2.64	0.7
MEQ external cues	2.62	0.52	2.59, 2.66	0.5
MEQ emotional eating	3.06	0.67	3.01, 3.10	0.7
MEQ distraction	2.94	0.57	2.90, 2.98	0.5

Abbreviations: CI, confidence intervals; MEQ, Mindful Eating Questionnaire; SD, standard deviation.

### 3.3 Associations with self‐reported BMI

Table 4 reports the fully adjusted regression models for each independent variable on self‐reported BMI when conducted separately and when added simultaneously in the same model (controlling for age, sex, education, household income, *n* = 691). Minimally adjusted models for separate and simultaneous models can be found in the Table S3. As the sample size with available data reduced from 830 to 691 when the additional confounding variables were added to the fully adjusted model, the minimally adjusted models were repeated with the sample of 691 (Table S4). Since the regression coefficients were similar between these two analyses, it was surmised that any differences were not driven by the reduced sample size in the fully adjusted model. Assumptions of skewness and kurtosis were met and the maximum VIF calculated was 1.96; as this does not exceed 4,^47^ it was surmised that multicollinearity is not an issue in this dataset.

**TABLE 4:** Fully adjusted regression models of self‐reported BMI (kg/m^2^).

Variables	Separate models			Simultaneous model		
	Beta	95% CI for beta	*p*	Beta	95% CI for beta	*p*
Episodic memory for recent eating	0.04	−0.04, 0.11	0.32	−0.01	−0.08, 0.06	0.77
Mindful eating (MEQ total)	−0.29	−0.36, −0.22	<0.001	−0.12	−0.20, −0.04	0.004
Distracted eating (composite)	0.1	0.02, 0.18	0.01	−0.007	−0.08, 0.07	0.85
Positive controls						
Restraint	−0.09	−0.17, −0.02	0.01	−0.04	−0.11, 0.03	0.23
Emotional eating (composite)	0.29	0.22, 0.36	<0.001	0.07	−0.02, 0.15	0.12
Disinhibition	0.38	0.32, 0.45	<0.001	0.30	0.21, 0.38	<0.001
Plate clearing	−0.01	−0.09, 0.07	0.76	0.03	−0.04, 0.11	0.34

*Notes*: Adjusted for age, sex, education, household income. Minimally adjusted models, controlling for age and sex can be found in Table S3. Beta = standardized coefficient.

Abbreviations: BMI, body mass index; CI, confidence intervals; MEQ, mindful eating questionnaire.

The separate regression models revealed that using episodic memory to think of your last meal before eating again was not associated with BMI. As noted above, this was not a common practice in this sample. Mindful eating, as measured by the MEQ, was associated with BMI, whereby the higher the total scores on this trait measure, the lower the BMI. In contrast, the composite measure of distracted eating was positively associated with BMI. From the “positive control” variables included, emotional and disinhibited eating were positively associated with BMI as expected, whereas plate clearing was not found to be associated with BMI, and restraint was negatively associated with BMI.

The relative associative strength of the variables of interest with self‐reported BMI was assessed using the simultaneous model (Table 4). All the psychological traits and eating behaviors measured in this study accounted for 23% of the variance in self‐reported BMI. This model confirmed that more frequent mindful eating was associated with lower self‐reported BMI, whereas disinhibited eating was associated with higher BMI. Minimally adjusted models (see Table S3) showed similar associations with BMI in both the separate and simultaneous models.

Planned exploratory analyses of the MEQ subscales and the questions about distracted eating are reported in Table S5.

### 3.4 Changes in eating behavior during first COVID‐19 lockdown

Data from participants living in the UK only were included in this analysis (*n* = 520) due to differing lockdown restrictions. A small number of participants reported having COVID‐19 symptoms within the previous 7 days of completing the survey (*n* = 10), with one participant having had a diagnosis of COVID‐19. Thirteen participants lived with others that had COVID‐19 symptoms, with a median (interquartile range) of 2 (0) people per household. The frequencies of responses given to the bespoke eating behavior questions when participants were asked to think about the past 7 days (during lockdown) can be found in Table 2.

Evidence was found for a lack of symmetry between the last 12 months and last 7 days in responses for most variables (McNemar–Bowker [3, 520], Table 5), suggesting a change in the frequency of the reported behaviors. One exception was memory for the last meal, for which no evidence for the change was found. Statistical evidence for change may not have been found for the memory variable (despite similar % change as other variables) due to the relative rarity of reporting this behavior. To further understand the pattern of data, the shift in responses (increase, decrease or no change between last 12 months and last 7 days) for each of the bespoke eating behavior variables is reported (Table 5). The majority of participants did not report any change in the frequency of engaging in each of the eating behaviors during lockdown compared with the previous 12 months. During lockdown, a higher percentage of participants decreased the reported frequency of eating mindfully focusing on taste, eating using a smart phone, or emotional and disinhibited eating.

**TABLE 5:** Change in reported frequency of each eating behavior measure in the last 7 days compared to the last 12 months.

Measure	Increase (%)	No change (%)	Decrease (%)	*p* Value
Memory for last meal	10.6	79.8	9.6	0.863
Mindful eating (texture)	6.7	82.7	10.6	0.012
Mindful eating (smell)	10.2	80.4	9.4	0.046
Mindful eating (taste)	8.3	73.3	18.5	<0.001
Distracted eating (gaming)	1.3	96.9	1.7	0.030
Distracted eating (watching TV)	7.5	81.3	11.2	<0.001
Distracted eating (while using smart phone)	6.7	76.0	17.3	<0.001
Positive controls				
Restrained eating	12.5	67.3	20.2	<0.001
Emotional eating (to feel better)	10.6	72.5	16.9	0.032
Emotional eating (when stressed)	4.2	68.1	27.7	<0.001
Emotional eating (when bored)	7.5	67.5	25.0	<0.001
Emotional eating (when sad)	4.4	61.2	34.4	<0.001
Disinhibition	10.4	66.2	23.5	<0.001
Plate clearing	6.2	85.6	8.3	0.028

*Note*: Participants located in the UK only (due to differing lockdown rules).

## 4 DISCUSSION

The main aim of this study was to investigate the relationship between cognitive controls over eating, namely episodic memory for recent eating, mindful eating and cognitive distraction, and self‐reported BMI for the first time in the same large sample. Contrary to previous reports that have shown a relationship between memory for recent eating and BMI,^7^ no evidence was found between the use of episodic memory in meal‐related decisions with BMI. While it is possible that this failure to replicate this association could be a genuine finding, there may be several explanations as to why this association was not found. First, only 12% of participants reported mostly or always recalling their previous meal before eating again. Secondly, the novel, bespoke measure of episodic memory for recent eating included in this study may not adequately capture the extent to which participants may utilize memory in eating‐related decisions and may therefore lack construct validity. It was a challenge to create an online measure to capture the rich dimensions of episodic memory processes without an accurate method for measuring actual intake. Future work should aim to improve this measure; for example, by asking participants to record their intake using an online dietary intake tool (e.g., intake24.co.uk) and then recall that same meal at a later timepoint.

Mindful eating, as measured by the total score on the MEQ,^31^ was negatively associated with BMI, such that the more regular participants reported using mindful techniques during eating, the lower their BMI, corroborating previous work.^27^, ^28^, ^29^, ^30^, ^31^ The exploratory analysis suggested that all the subscales of the MEQ were associated with BMI with the exception of the external subscale. However, the internal consistency of the external and distracted eating subscales of the MEQ fell below the accepted range.^46^ Closer inspection of the items in the MEQ subscales suggests that the disinhibition subscale (inability to stop eating when one is full) measures disinhibited eating and the emotional eating subscale (e.g., eating to feel better when sad) measures emotional eating, rather than a unique construct of mindful eating per se. Both subscales were reversed scored, reporting a lack of disinhibited or emotional eating rather than the absence of mindful eating. Indeed, the bespoke measures of disinhibited and emotional eating included in this study predict the reverse relationship with BMI as to the MEQ subscale comparators. By contrast, the awareness subscale of the MEQ refers to the extent to which individuals are conscious of the taste, smell, and appearance of the food being consumed, which more closely aligns with the definition of mindful eating/mindfulness.^48^, ^49^ Strong evidence for a negative relationship between the awareness subscale and BMI was found, suggesting that future work should investigate this relationship further with other mindful eating instruments, such as the recently validated Mindful Eating Inventory (MEI^48^). The MEI measures all facets of mindfulness as applied to eating‐specific contexts, incorporating aspects included in other validated scales.^48^

It is noted that the data presented here are cross‐sectional and it is not possible to infer causality from them. However, these data suggest that mindful eating might represent a risk factor for overweight and obesity. Future research should evaluate whether mindful eating demonstrates a prospective association with body weight. It is noted that mindfulness‐based interventions have previously been applied in weight loss interventions with mixed results.^49^, ^50^ Tapper (2022) argues that future research needs to understand mechanisms of action to determine what mindful eating strategies are most likely to be helpful and whether certain individuals should be targeted. It is beyond the scope of this paper to consider all the potential mechanisms of action. However, readers are directed to the “Potential Mechanisms of Action” section of this recent narrative review.

Distractedly eating was positively associated with BMI in this sample, consistently across the composite measure, individual measures of eating while gaming or using a smart phone, and the MEQ distraction subscale. The composite score of distraction included in the simultaneous model did not remain associated with BMI, suggesting that the component of BMI explained by distracted eating may be shared with the other variables, such as mindful eating as discussed above, but also there could be an element of disinhibited/uncontrolled eating while distracted. Contrary to previous reports,^15^, ^51^ eating while watching television was not associated with BMI. It is possible that mobile phone use and playing a computer game while eating are more “distracting” or cognitively engaging activities compared to watching TV.^52^ Indeed, “attention to TV” has been shown to be the most important factor over time spent watching.^53^

With regard to the positive controls, that have established relationships with BMI, disinhibition and emotional eating were positively associated with BMI.^25^ Contrary to previous reports,^25^ a negative association was found between the measure of restraint and BMI and note that other studies have also found weak evidence for this relationship.^54^, ^55^ It is plausible that the wording of the question (How often do you avoid eating tempting foods?) could be a measure of successful restriction, rather than the intention to restrain, which is often linked to disinhibition.^56^ The failure to replicate the association between plate clearing and BMI could be due to the relatively low frequency of reported plate clearing in this sample. Robinson et al.,^35^ quantified the extent to which individuals leave food on the plate—only 13.2% reported often or always leaving food on the plates, whereas 66.9% in this sample reported mostly or always leaving food on the plate. Disinhibition (bespoke measure) was the only variable that remained positively associated with BMI in the simultaneous model, and indeed showed the strongest overall association with BMI. These findings corroborate previous evidence that regular disinhibited eating is a strong predictor of higher BMI,^57^ and here this is demonstrated over and above other cognitive controls and traits relating to eating behavior.

Finally, of the four potentially confounding variables included in the fully adjusted simultaneous regression, only age and sex showed an association with self‐reported BMI, whereas household income and highest level of education did not (see Table S11 for relevant statistics). Although this pattern of associations with BMI differs from previous similar reports, such as Robinson et al.^35^ who found a relationship between BMI and age, gender and education but not income, it is likely that these relationships will be idiosyncratic to the demographics of the specific sample included in the study (see Limitations section below).

The timing of this study allowed a concurrent investigation into potential changes in eating behavior, including cognitive controls of eating, during the COVID‐19 lockdown in the UK (April–May 2020). Evidence for change was found in nearly all eating behaviors (with the exception of episodic memory for recent eating), between the previous 12 months and during lockdown. However, for the large majority of these measures, the relative proportion of those who increased or decreased the frequency of those behaviors was similar. Based on the set threshold (reported in *Before and after lockdown analyses, Statistical analysis section*), consistent change was seen in the frequency of certain eating behaviors, namely a reduction in eating while using a smart phone, emotional and disinhibited eating, and eating mindfully focusing on taste. It is possible that due to being confined to the home, where screen‐time has generally increased,^58^ participants may have felt less inclined to engage with these distractions during their evening meal. This study was a self‐selecting sample, and therefore, it was perhaps more likely that those individuals who participated had more time and were living in less stressful circumstances. Other studies have measured the impact of the pandemic on psychological traits related to eating, including craving control,^37^ appetitive drive,^38^ and self‐control and disinhibited eating.^59^ Specifically, while emotional eating *decreased* overall in the sample, others have found during the pandemic that higher emotional eating was associated with higher BMI^39^, ^60^ and maladaptive coping strategies.^60^

While it may seem counterintuitive that the majority of eating behaviors did not show overall change, it should be considered that experiences differed widely during this period. Despite the stressful circumstances, there was less commuting with many on furlough, so some people will have had more time to consider food‐related activities^61^ and coped with the situation well,^62^ while others with pre‐existing conditions may have found the experience more stressful.^63^ Indeed, those with lower trait self‐control, reported greater disinhibited eating possibly due to levels of negative affect.^59^ This variability in experience (perhaps relating to work, childcare, and health status) is reflected in this data, given the mixed pattern of results.

With consideration of the limitations of this study, it should be noted that the recruited sample was relatively highly educated and had lower rates of overweight and obesity than the average British population.^64^, ^65^ Specifically, the comparison of this sample with the most recent figures from The Health Survey for England 2021 suggests that the sample did have lower rates of obesity (15.5%) than the average British population (25.9%). Regarding the highest level of education attained, while this sample had a similar percentage of those attaining level 4 (Degree, 35.9%) to figures from Census 2021 (level 4 33.8%), a lower percentage in this sample reported no qualifications (1.2% compared to 18.2% in Census 2021). It is possible therefore that lower rates of overweight and obesity in this sample could explain discrepancies in findings between this and aforementioned study in this area as discussed above, and may therefore limit the generalizability of the findings. A second consideration is that the questionnaire design employed in this study required retrospective recall of eating behaviors over the past 12 months compared to the past 7 days. It is possible that the unusual “lockdown” circumstances at the time of data collection may have influenced participants' memory of previous behaviors over the last 12 months. However, we note that retrospective reports have been successfully employed in previous research over significantly longer time periods than 12 months (e.g., recall of childhood experience) and found to be reliable.^66^, ^67^

## 5 CONCLUSION

Robust relationships between self‐reported BMI and cognitive controls over eating have been reported here, with a negative relationship with mindful eating and a positive relationship with distracted eating. The predicted positive associations between self‐reported BMI and the positive controls (restrained eating, emotional eating and disinhibited eating) were also observed. However, we failed to replicate a positive association between self‐reported BMI and plate clearing in our sample. Importantly, in a simultaneous model, it was possible to compare the relative strength of the association of each of these variables with BMI. Mindful eating demonstrated a small effect size relationship with BMI, and disinhibited eating showed a medium effect size relationship.^68^ Strategically, these eating behaviors could be useful targets for future behavioral interventions, such as reducing disinhibition through response inhibition training^69^ or increasing mindful eating.^49^ However, it is noted that the findings reported here are cross‐sectional and causality cannot be inferred. Future research should evaluate whether mindful eating demonstrates a prospective association with body weight and should consider mechanisms of action.

## AUTHOR CONTRIBUTIONS

Elanor C. Hinton, Victoria Beesley and Danielle Ferriday conceived and designed the study. Victoria Beesley set up the survey and conducted the data collection. Elanor C. Hinton and Victoria Beesley conducted the data analysis, under the guidance of Sam D. Leary. All authors provided an interpretation of the data. Elanor C. Hinton, Victoria Beesley and Danielle Ferriday wrote the first draft of the paper, and all authors reviewed and approved the final version of the manuscript.

## CONFLICT OF INTEREST STATEMENT

Dr Hinton is employed by both the University of Bristol as a Research Fellow (through which she completed this work) and also on a part‐time basis with Oxford Medical Products as a Clinical Studies Manager. Please note that the work reported in this paper was conducted fully independently and before the role with the company began. The other authors have no declarations of interest.

## Supporting information

Supporting Information S1
