# Supplementary material for: Associations between body mass index and episodic memory for recent eating, mindful eating, and cognitive distraction: A cross‐sectional study
Source: Obes Sci Pract. 2024 Jan 4;10(1):e728. doi: 10.1002/osp4.728 (PMC10768738; doi:10.1002/osp4.728)
Supplement: Supplementary file 1 — Supporting Information S1 [file OSP4-10-e728-s001.docx]

**Supporting Information**

**Associations between body mass index and episodic memory for recent eating, mindful eating, and cognitive distraction: a global cross-sectional study in adults.**

Elanor C Hinton^1*^, Victoria Beesley^2^, Sam D Leary^1^, Danielle Ferriday^1,2^

^1^NIHR Bristol Biomedical Research Centre Diet and Physical Activity Theme, University of Bristol, Bristol, U.K.

^2^Nutrition and Behaviour Unit, School of Psychological Science, University of Bristol, Bristol, U.K.

**Table S1:** Sociodemographic questions included in the survey

| Survey Item | Response Options |
| --- | --- |
| 1.What is your sex? | Male, female, prefer not to say, other (please specify) |
| 2. What is your date of birth? | Day, month, year |
| 3. How would you describe yourself (ethnicity)?  (Question taken from ALSPAC†) | White, Gypsy or Traveller, Black or Black British-Caribbean, Black or Black British-African, Asian or Asian British-Indian, Asian or Asian British-Pakistani, Asian or Asian British-Bangladeshi, Chinese, Mixed-White and Black Caribbean, Mixed-White and Black African, Mixed-n White and Asian, prefer not to say, Not known, Other (please specify) |
| 4. What is your nationality? | Free text response |
| 5. What is the highest level of education you have completed? | None, GCSEs or equivalent, A-Levels or equivalent, Degree, Higher Degree, other, prefer not to say |
| 6. How many others live in your household?  (This was asked in a subsample of 699) | 0, 1, 2, 3, 4, 5, 6, 7, 8, 9, 10, 11, 12, 13, 14, 15, more than 15 |
| 7. On average, about how much is your take-home household income per month? | less than £899, £900-£1149, £1150-£1549, £1550-£1849, £1850-£2099, £2100-£2399, £2400-£2799, £2800-£3399, £3400-£4000, £4001 or more, prefer not to say. |
| 8. In which country are you currently living? | Free text response |
| 9. What is your height? | Imperial (feet and inches) or Metric (metres and centimeters) |
| 10. What is your weight? | Imperial (stone and pounds) or Metric (kilograms) |
| 11. Have you ever been diagnosed with an eating disorder? | Yes/No |

**†** <https://alspac-explore.bristol.ac.uk/mica-web/mica/variable/t%3At1300%3ACollected>

**Table S2:** Mean appetite ratings and state questions at time of questionnaire completion

| Measure | Mean | Standard deviation | 95% CI (lower, upper) |
| --- | --- | --- | --- |
| How hungry do you feel right now? (100mm VAS) | 26.5 | 27.5 | 24.7, 28.4 |
| How thirsty do you feel right now? (100mm VAS) | 37.7 | 28.5 | 35.8, 39.7 |
| How full does your stomach feel right now? (100mm VAS) | 50.7 | 28.0 | 48.8, 52.5 |
| Time since last eaten (minutes) | 257.5 | 317.4 | 236.1, 279.0 |
| Time since last drank (minutes) | 135.1 | 269.4 | 117.0, 153.3 |
| Last meal vividness (100mm VAS) | 84.5 | 23.0 | 83.0, 86.1 |
| Last drink vividness (100mm VAS) | 83.9 | 25.21 | 82.2, 85.5 |

**Table S3:** Minimally adjusted regression models (separate and simultaneous) for each eating behaviour variable of self-reported BMI with n=830

|  | **Separate models** | |  | **Simultaneous model** | |  |
| --- | --- | --- | --- | --- | --- | --- |
| **Variables** | **Beta** | **95% CI for Beta** | **p** | **Beta** | **95% CI for Beta** | **p** |
| Episodic memory for recent eating | 0.01 | -0.05, 0.08 | 0.71 | -0.04 | -0.11, 0.02 | 0.17 |
| Mindful eating (MEQ total) | -0.28 | -0.34, -0.22 | <0.000 | -0.11 | -0.18, -0.04 | 0.004 |
| Distracted eating (composite) | 0.107 | 0.04, 1.8 | 0.003 | 0.02 | -0.05, 0.09 | 0.56 |
| **Positive controls:** |  |  |  |  |  |  |
| Restraint | -0.07 | -0.14, -0.01 | 0.03 | -0.02 | -0.08, 0.04 | 0.50 |
| Emotional eating (composite) | 0.29 | 0.23, 0.35 | <0.001 | 0.09 | 0.01, 0.17 | 0.03 |
| Disinhibition | 0.36 | 0.30, 0.42 | <0.001 | 0.27 | 0.19, .34 | <0.001 |
| Plate clearing | -0.02 | -0.09, 0.05 | 0.55 | 0.02 | -0.04, 0.08 | 0.50 |

**Table S4:** Minimally adjusted regression models for each hypothesised predictor of self-reported BMI with n=691

|  | **Separate models** | |  | | | **Simultaneous model** | |  | |  |
| --- | --- | --- | --- | --- | --- | --- | --- | --- | --- | --- |
| **Variables** | **Beta** | **95% CI for Beta** | | **p** | **Beta** | | **95% CI for Beta** | | **p** | |
| Episodic memory for recent eating | 0.04 | -0.04, 0.11 | | 0.35 | -0.01 | | -0.08, 0.06 | | 0.73 | |
| Mindful eating (MEQ total) | -0.29 | -0.36, -0.22 | | <0.001 | -0.12 | | -0.20, -0.04 | | 0.003 | |
| Distracted eating (composite) | 0.09 | 0.02, 0.17 | | 0.02 | -0.01 | | -0.09, 0.06 | | 0.76 | |
| **Positive controls:** |  |  | |  |  | |  | |  | |
| Restraint | -0.09 | -0.17, -0.02 | | 0.01 | -0.04 | | -0.11, 0.03 | | 0.24 | |
| Emotional eating (composite) | 0.29 | 0.22, 0.36 | | <0.001 | 0.07 | | -0.02, 0.15 | | 0.12 | |
| Disinhibition | 0.38 | 0.32, 0.45 | | <0.001 | 0.29 | | 0.21, 0.38 | | <0.001 | |
| Plate clearing | -0.01 | -0.09, 0.07 | | 0.81 | 0.04 | | -0.03, 0.11 | | 0.30 | |

*A priori exploratory analyses of MEQ and distracted eating*

Planned exploratory analyses of the MEQ subscales (Table S5) indicated that the negative association with BMI reported above was largely driven by the disinhibition and emotional eating subscales (note higher scores on the MEQ subscales indicate higher mindful eating, therefore less disinhibited and emotional eating), with no association between the external subscale and BMI.

The second set of planned exploratory regressions of the individual questions comprising distracted eating (Table S5) suggested that the positive association between distracted eating and BMI was driven by response to the eating whilst using a smart phone or whilst gaming. The pattern of results was similar for the minimally and fully adjusted models for both sets of explanatory analyses.

**Table S5:** Planned exploratory analyses of MEQ subscales and distracted eating variables on self-reported BMI (kg/m^2^)

|  | **Minimally adjusted model*** | |  | **Fully adjusted model†** | |  |
| --- | --- | --- | --- | --- | --- | --- |
| **Individual regressions for each variable:** | **Beta** | **95% CI for Beta** | **p** | **Beta** | **95% CI for Beta** | **p** |
| **MEQ subscales:** |  |  |  |  |  |  |
| Awareness | -0.13 | -0.19, -0.06 | <0.001 | -0.14 | -0.21, -0.07 | <0.001 |
| Distraction | -0.10 | -0.17, -0.03 | 0.003 | -0.12 | -0.19, -0.05 | 0.002 |
| Disinhibition | -0.27 | -0.33, -0.21 | <0.001 | -0.28 | -0.036, -0.21 | <0.001 |
| Emotional | -0.23 | -0.30, -0.17 | <0.001 | -0.25 | -0.32, -0.17 | <0.001 |
| External | -0.05 | -0.12, 0.02 | 0.17 | -0.04 | -0.12, 0.04 | 0.29 |
| **Distracted eating (individual questions):** |  |  |  |  |  |  |
| Eating whilst using smart phone | 0.07 | 0.00, 0.14 | 0.04 | 0.08 | 0.01, 0.17 | 0.04 |
| Eating whilst gaming | 0.11 | 0.04, 0.18 | 0.003 | 0.10 | 0.02, 0.18 | 0.01 |
| Eating whilst watching TV | 0.04 | -0.02, 0.11 | 0.20 | 0.03 | -0.05, 0.10 | 0.47 |
| **Emotional eating (individual questions):** |  |  |  |  |  |  |
| How often do you eat: |  |  |  |  |  |  |
| when you are sad? | 0.22 | 0.15, 0.28 | <0.001 | 0.22 | 0.15, 0.30 | <0.001 |
| when you are stressed? | 0.25 | 0.19, 0.32 | <0.001 | 0.25 | 0.18, 0.32 | <0.001 |
| when you are bored? | 0.17 | 0.10, 0.24 | <0.001 | 0.18 | 0.11, 0.25 | <0.001 |
| to make yourself feel better? | 0.18 | 0.11, 0.25 | <0.001 | 0.19 | 0.11, 0.26 | <0.001 |

*adjusted for age and sex; †adjusted for age, sex, education and household income

**Figure S1:** Distribution of participants based on global location.

**Additional analyses to address pre-registered hypotheses**


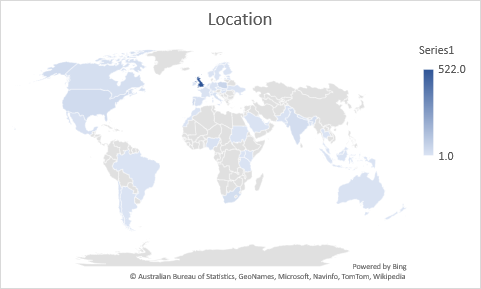


No. of participants

(1) Adult Eating Behaviour Questionnaire measures

Food approach and food avoidance was measured using the relevant appetitive traits from the Adult Eating Behaviour Questionnaire (AEBQ; [1]), a 35-item questionnaire which measures four ‘food approach’ constructs: hunger, food responsiveness, emotional overeating, enjoyment of food, and four ‘food avoidance’ subscales: satiety responsiveness (3 items), emotional undereating (5 items), food fussiness (5 items including 3 reverse coded items), and slowness in eating (4 items including 1 reverse coded item). All items are scored on a 5-point Likert-type scale ranging from 1 (‘Strongly Disagree’) to 5 (‘Strongly Agree’), with higher scores signifying higher trait levels. According to Hunot et al. (2016), the AEBQ is a valid measure of appetitive traits with good internal consistency (Cronbach’s a = 0.7). The scores on the food avoidance AEBQ subscales were combined into a single measure (mean of satiety responsiveness, food fussiness, emotional undereating, and slowness in eating). The AEBQ was included as a separate section of the online questionnaire between the MEQ and the COVID-19 sections.

In the pre-registration, it was hypothesised that:

- Individuals with a higher self-reported BMI will have a higher score on food approach subscales of the AEBQ.
- Individuals with a higher self-reported BMI will have a lower score on food avoidance subscales of the AEBQ.

**Table S6:** Mean (SD) and reliability of the AEBQ

| Measure | Mean | Standard deviation | 95% CI (lower, upper) | Internal consistency (a) |
| --- | --- | --- | --- | --- |
| AEBQ Total | 3.0 | 0.3 | 3.0, 3.0 | 0.69 |
|  |  |  |  |  |
| *Food avoidance subscales:* |  |  |  |  |
| Satiety Responsiveness | 2.6 | 0.8 | 2.6, 2.7 | 0.7 |
| Emotional Under-eating | 3.1 | 0.9 | 3.0, 3.1 | 0.9 |
| Food Fussiness | 3.3 | 0.4 | 3.3, 3.4 | 0.9 |
| Slowness in Eating | 2.9 | 0.6 | 2.9, 3.0 | 0.9 |
|  |  |  |  |  |
| *Food avoidance subscales:* |  |  |  |  |
| Hunger | 3.1 | 0.7 | 3.1, 3.2 | 0.68 |
| Food Responsiveness | 3.2 | 0.8 | 3.1, 3.2 | 0.72 |
| Emotional Over-Eating | 2.6 | 0.9 | 2.6, 2.7 | 0.87 |
| Enjoyment of Food | 4.1 | 0.8 | 4.1, 4.2 | 0.84 |

**Table S7:** Regression analyses for AEBQ food approach and food avoidance with self-reported BMI (kg/m^2^)

|  | **Minimally adjusted model*** | | | **Fully adjusted model**† | |  |
| --- | --- | --- | --- | --- | --- | --- |
| **Variables** | **Beta** | **95% CI for Beta** | **p** | **Beta** | **95% CI for Beta** | **p** |
| Food approach | 0.19 | 0.12, 0.25 | <0.001 | 0.21 | 0.13, 0.28 | <0.001 |
| Food avoidance | -0.16 | -0.23, -0.10 | <0.001 | -0.17 | -0.24, -0.09 | <0.001 |

*adjusted for age and sex; †adjusted for age, sex, education and household income

(2) Additional bespoke variables (hoarding, eating in the absence of hunger and eating with others) and self-reported BMI

As part of a broader Master’s thesis, several bespoke questions were included in the original questionnaire but were not reported in the main paper. In the pre-registration, it was hypothesised that:

- Individuals who often keep their cupboards full will have a higher self-reported BMI, compared to individuals who do not keep their cupboards full often. [measured with the question: How often do you keep your cupboards full? Included within the usual eating section (section 3)]
- Individuals who report often eating even though they are full will have a higher self-reported BMI, compared to individuals who do not report often eating even though they are full. [measured with the question: How often do you eat even though you are not hungry? Included within the usual eating section (section 3)]

Table S8 reports the separate regressions for each question with BMI, both minimally and fully adjusted as per the results reported elsewhere in the manuscript.

**Table S8:** Regression analyses for hoarding, eating in the absence of hunger with self-reported BMI (kg/m^2^)

|  | **Minimally adjusted*** | | | **Fully adjusted**† | | |
| --- | --- | --- | --- | --- | --- | --- |
| **Variables** | **Beta** | **95% CI for Beta** | **p** | **Beta** | **95% CI for Beta** | **p** |
| Hoarding | 0.03 | -0.04, 0.10 | 0.43 | 0.03 | -0.05, 0.10 | 0.49 |
| Eating in the absence of hunger | 0.18 | 0.12, 0.25 | <0.001 | 0.19 | 0.12, 0.26 | <0.001 |

In the original questionnaire we included a fourth question to measure potential distracted eating: how often do you eat with others? (Included within the usual evening meal section (section 4). We removed this data from the calculation of composite score for distraction as it was subsequently decided that this question tapped into the concept of social facilitation of eating rather than distraction. Table S9 reports the minimal and fully adjusted regression with BMI for this variable.

**Table S9:** Regression analyses for social facilitation with self-reported BMI (kg/m^2^)

|  | **Minimally adjusted*** | |  | **Fully adjusted**† | |  |
| --- | --- | --- | --- | --- | --- | --- |
| **Variables** | **Beta** | **95% CI for Beta** | **p** | **Beta** | **95% CI for Beta** | **p** |
| Eat with others | 0.01 | -0.06, 0.07 | 0.84 | 0.02 | -0.05, 0.10 | 0.59 |
|  |  |  |  |  |  |  |

(3) Additional bespoke variable pre-post lockdown

In the pre-registered questionnaire, it was hypothesised that:

- Individuals will hoard food and keep their cupboards full more during the COVID-19 pandemic compared to before the pandemic (question included in section 5 of online questionnaire).

The results from the McNemar-Bowker test are shown in Table S10.

**Table S10:** Percentage of participants who reported changing the frequency of hoarding (increase, decrease or no change) in the last 7 days compared to usual behaviour over the last 12 months

| Measure | Increase (%) | | No change (%) | | Decrease (%) | p value |
| --- | --- | --- | --- | --- | --- | --- |
| Hoarding | 9.4 | 79.6 | | 11.0 | | 0.090 |

**Table S11:** Associations between confounding variables and self-reported BMI (kg/m^2^) from the fully adjusted simultaneous regression model.

|  | **Regression** | |  |
| --- | --- | --- | --- |
| **Variables** | **Beta** | **95% CI for Beta** | **p** |
| Sex | -0.08 | -0.15, 0.01 | 0.029 |
| Age | 0.29 | 0.22, 0.37 | <0.001 |
| Education | 0.01 | -0.08, 0.06 | 0.821 |
| Household income | 0.02 | -0.05, 0.10 | 0.511 |

References:

1. Hunot, C., et al., *Appetitive traits and relationships with BMI in adults: Development of the Adult Eating Behaviour Questionnaire.* Appetite, 2016. **105**: p. 356-363.
